# Ecography guided puncture vs traditional puncture lancing: benefits for patients at risk in number of attempts to giving comfortable area

**DOI:** 10.1186/2036-7902-4-S1-A30

**Published:** 2012-12-18

**Authors:** Estefania Ruiz Chacon, Jordi Moreno Citoler, Ramon Nogué Bou, Joan Pere Fabregat

**Affiliations:** 1 Hospital Montserrat Lleida, Servicio de Urgencias y Cuidados Intermedios, Spain

## Aim

The purpose of this preliminary study is to verify the venipuncture technical using the ecography scanner and compared to the traditional puncture and how this first is beneficial for the patient decreasing the number of attempts and saving the affected puncture area.

Inclusion criteria: Including the patient risk and his pathology study: obesity, multipuncturation, respiratory pathology, cardiovascular pathology, patients with warfarina treatment; and convenient area of puncture, defining it as the area between radio-ulnar 1/3 distal and proximal radio-ulnar 1/3.

## Method

A prospective study in two groups of patients with the above-mentioned pathologies: 50 patients receiving traditional puncture and 50 patients receiving ecography guided puncture.

## Results

Traditional puncture gets 56% success on the first try, while with ecography guided puncture the success increases to 72%.

Traditional puncture on the second attempt we obtain a 34% and with ecography guided puncture we have a 26% of success.

We got, with the third attempt in traditional puncture, a 12% of success and through ecography guided puncture we have a 2% of success.

Fourth attempt to puncture through the traditional method is done in 2% patients while ecography guided puncture method the result is 0%.

## Conclusion

The percentage of success in the first ecography guided puncture is greater than the traditional method.

The percentage of success increases through the ecography puncture method meanwhile increases in the number of attempts.

The area where to place venous catheter is relevant to consider area of comfortable puncture for the patient described above.

With the same group of patients the choice of convenient area of puncture site was 92% entering in the above described area between distal radio-ulnar 1/3 and proximal radio-ulnar 1/3. The 8% remaining was in different areas of the upper limb.

